# SF3B1 mutation in pancreatic cancer contributes to aerobic glycolysis and tumor growth through a PP2A–c‐Myc axis

**DOI:** 10.1002/1878-0261.12970

**Published:** 2021-05-03

**Authors:** Jian‐Yu Yang, Yan‐Miao Huo, Min‐Wei Yang, Yang Shen, De‐Jun Liu, Xue‐Liang Fu, Ling‐Ye Tao, Rui‐Zhe He, Jun‐Feng Zhang, Rong Hua, Shu‐Heng Jiang, Yong‐Wei Sun, Wei Liu

**Affiliations:** ^1^ Department of Biliary‐Pancreatic Surgery, Ren Ji Hospital, School of Medicine Shanghai Jiao Tong University Shanghai China; ^2^ State Key Laboratory of Oncogenes and Related Genes Shanghai Cancer Institute School of Medicine Ren Ji Hospital Shanghai Jiao Tong University China

**Keywords:** pancreatic ductal adenocarcinoma, PP2A, SF3B1, splicing factor, Warburg effect

## Abstract

Hot spot gene mutations in splicing factor 3b subunit 1 (*SF3B1*) are observed in many types of cancer and create abundant aberrant mRNA splicing, which is profoundly implicated in tumorigenesis. Here, we identified that the SF3B1 K700E (SF3B1^K700E^) mutation is strongly associated with tumor growth in pancreatic ductal adenocarcinoma (PDAC). Knockdown of SF3B1 significantly retarded cell proliferation and tumor growth in a cell line (Panc05.04) with the SF3B1^K700E^ mutation. However, SF3B1 knockdown had no notable effect on cell proliferation in two cell lines (BxPC3 and AsPC1) carrying wild‐type SF3B1. Ectopic expression of SF3B1^K700E^ but not SF3B1^WT^ in SF3B1‐knockout Panc05.04 cells largely restored the inhibitory role induced by SF3B1 knockdown. Introduction of the SF3B1^K700E^ mutation in BxPC3 and AsPC1 cells also boosted cell proliferation. Gene set enrichment analysis demonstrated a close correlation between SF3B1 mutation and aerobic glycolysis. Functional analyses showed that the SF3B1^K700E^ mutation promoted tumor glycolysis, as evidenced by glucose consumption, lactate release, and extracellular acidification rate. Mechanistically, the SF3B1 mutation promoted the aberrant splicing of PPP2R5A and led to the activation of the glycolytic regulator c‐Myc via post‐translational regulation. Pharmacological activation of PP2A with FTY‐720 markedly compromised the growth advantage induced by the SF3B1^K700E^ mutation *in vitro* and *in vivo*. Taken together, our data suggest a novel function for SF3B1 mutation in the Warburg effect, and this finding may offer a potential therapeutic strategy against PDAC with the SF3B1^K700E^ mutation.

## Graphical abstract

SF3B1 mutations have been previously implicated in tumorigenesis. Here, we investigate the role of SF3B1^K700E^ mutation in pancreatic ductal adenocarcinoma (PDAC). SF3B1^K700E^ led to aberrant splicing of PPP2R5A, coupled with c‐Myc activation higher aerobic glycolysis rate and growth advantage of tumor cells. Taken together, our data describe a novel function for SF3B1 ^K700E^ mutations in the Warburg effect. Inhibition of SF3B1 ^K700E^ mutation may potentially serve as a novel therapeutic strategy for patients with PDAC.

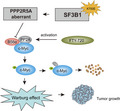

**Abbreviations**
CHX: cycloheximide
ECAR: extracellular acidification rate
FBS: fetal bovine serum
GTEx: Genotype‐Tissue Expression
MDS: myelodysplastic syndrome
PDAC: pancreatic ductal adenocarcinoma
pre‐mRNA: precursor messenger RNA
SF3B1: splicing factor 3b subunit 1
TCGA: The Cancer Genome Atlas

## 1 Introduction

Pancreatic ductal adenocarcinoma (PDAC) is one of the most malignant and deadly solid tumors, exhibiting an extremely poor prognosis [1]. In recent years, the incidence of PDAC has shown a rapid upward trend and PDAC ranks sixth and seventh among males and females for cancer‐related deaths, respectively [2]. Therefore, PDAC is a serious threat to lives and health. Deciphering the molecular mechanism of PDAC pathogenesis and identifying effective therapeutic targets are of great importance in pancreatic cancer research.

RNA splicing is the biological process of precursor messenger RNA (pre‐mRNA) by removing introns from the spliceosome to form mature mRNA. Pre‐mRNA produces different types of mRNA under different splicing methods [3]. This process is referred to as alternative splicing, which is considered to be an important mechanism to increase the complexity of the proteome. It maintains system homeostasis during the development of different types of cells in the organism and responds to external stimuli [4, 5, 6, 7]. The alteration and formation of aberrant alternative splicing play an important role in the occurrence and development of diseases, especially cancers [8, 9, 10, 11].

The splicing factor is responsible for the splicing process. Alternative splicing induced by abnormal expression and/or mutations of splicing factors is an important molecular feature of tumorigenesis [12, 13, 14, 15]. The abnormal expression of splicing factors can induce changes in many key tumor‐related genes, such as RON, BIN1, S6K1, MNK2, BIM, and BCL‐x, which in turn affects malignant phenotypes including cell proliferation, metastasis, chemoresistance, and other processes [16, 17, 18, 19]. Notably, mutations in splicing factors play an important role in the occurrence and development of tumors, especially in hematological tumors. Kim and Shirai et al. reported that mutations in the splicing factors SRSF2 and U2AF1 regulate the alternative splicing of related genes by changing the activity of their sequence‐specific RNA binding sites and eventually lead to the occurrence of hematological tumors [20, 21].

The splicing factor SF3B1 is a core part of the U2 small nuclear ribonucleoprotein complex. Normally, SF3B1 is essential for the proper selection of 3′ acceptor sequences in pre‐mRNA splicing reactions. In cancers, SF3B1 mutations generate a neomorphic protein that disrupts RNA splicing and leads to the downregulation of mRNA from hundreds of affected genes [22, 23, 24, 25]. Interestingly, it has been reported that SF3B1 is frequently mutated in many types of human cancers, including myelodysplastic syndrome (MDS), chronic lymphocytic leukemia, breast cancer, uveal melanoma, and PDAC [26, 27, 28, 29, 30]. Recently, emerging studies have documented SF3B1‐mediated splicing alterations and the downstream cellular processes in its oncogenic activities [22, 28, 31]. To date, the potential oncogenic roles and underlying molecular mechanisms of SF3B1 mutation in PDAC have not been identified.

In this study, by loss‐of‐function and gain‐of‐function studies, we demonstrated that SF3B1 K700E mutation favored *in vitro* cell proliferation and *in vivo* tumor growth in pancreatic cancer cells. Gene set enrichment analysis of PDAC samples from The Cancer Genome Atlas (TCGA) revealed that the SF3B1 mutation was closely associated with the Warburg effect. Further mechanistic studies identified that the SF3B1 K700E mutation resulted in aberrant splicing of PPP2R5A and led to an increase in c‐Myc expression, which ultimately promoted the Warburg effect and tumor growth in PDAC.

## 2 Materials and methods

### 2.1 Bioinformatic analysis

For determining SF3B1 expression, the online GEPIA2 database [32] was searched. GEPIA2 provides an overview of TCGA and The Genotype‐Tissue Expression (GTEx) project. We utilized GEPIA2 to analyze the expression profile and prognostic value of the SF3B1 gene in pancreatic cancer. For SF3B1 mutation and copy number analysis, the cBioPortal database (http://www.cbioportal.org/) was used.

### 2.2 Cell lines and reagents

Human pancreatic cancer cell lines including AsPC1, Capan‐2, BxPC3, Capan‐1, CFPAC‐1, MiaPaCa‐2, SW1990, PANC‐1, Panc05.04, and the nonmalignant HPDE cell line were preserved in Ren Ji Hospital. Detailed cell line information was provided in Table S1. All cells were maintained in the culture medium as suggested by protocols by American Type Culture Collection. Briefly, cells were cultured with 10% (v/v) FBS (Gibco, New York, NY, USA) and 1% (v/v) penicillin–streptomycin (Sigma‐Aldrich, St. Louis, MO, USA) at 37 °C and 5% CO_2_ condition. PP2A activator FTY‐720 was purchased from Selleck (S5002, Shanghai, China). The concentration of FTY‐720 used in this study is 5 μm. For colony formation assay, FTY‐720 was administrated every 48 h; for western blotting and measurement of tumor glycolysis, FTY‐720 was administrated for 24 h. And FTY‐720 was administered after the cells were seeded for 12 h.

### 2.3 Plasmids and cell transfection

Two specific short hairpin RNAs (shRNAs) against SF3B1 were synthesized by GenePharma (Shanghai, China). HEK293T cells were seeded and transfected along with a three plasmid system (pPACKH1‐GAG, pPACKH1‐REV, and pVSV‐G) using Lipofectamine 2000 (Invitrogen, Carlsbad, CA, USA) following the guidelines by the manufacturer. Cell supernatants containing viral particles were harvested at 72 h after transfection and filtered through 0.45‐μm filters. Panc05.04 cells were cultured in a 6‐well plate at a density of 3 × 10^5^ per well and infected with 0.5 mL recombinant lentivirus in the presence of 5 μg·mL^−1^ polybrene (H9268; Sigma‐Aldrich). Next, sh‐SF3B1‐expressing cells were selected with puromycin (2 μg·mL^−1^; Gibco) for 1–2 weeks. The knockdown efficiency of SF3B1 was verified by western blotting. The K700E mutant SF3B1 plasmid was a gift from Marc‐Henri Stern (Department of Genetics and Biology of Cancers, INSERM U830, Institute Curie, PSL Research University). Full‐length wild‐type SF3B1 and K700E mutant SF3B1 were subcloned into pCDH‐CMV‐MCS‐EF1‐Puro vector to generate SF3B1^WT^ and SF3B1^K700E^ overexpression plasmids. Plasmid transfection was performed in Panc05.04 cells using 500 ng of plasmid construct and Lipofectamine 2000 reagent (Invitrogen) according to the manufacturer’s instructions. The transfection efficiency was verified before subsequent cellular experiments.

### 2.4 Western blotting analysis

Western blot analyses of whole‐cell protein lysates were performed essentially as reported previously [33]. In brief, protein lysates were quantified and separated by SDS/PAGE and transferred to polyvinylidene difluoride membranes which were then probed with one of the following primary antibodies, SF3B1 (diluted at 1 : 2000; Abcam, Hanzhou, China, ab172634), c‐Myc (diluted at 1 : 1000; Cell Signaling Technology, Shanghai, China, #5605), p‐c‐Myc (S62) (diluted at 1 : 1000; Cell Signaling Technology, #13748), PPP2R5A (diluted at 1 : 2000; Abcam, ab89621), and β‐actin (diluted at 1 : 2000; Abcam, ab8226). Finally, membranes were supplemented with species‐specific secondary antibodies, and immunoreactivity was detected by Odyssey imaging system (LI‐COR Biosciences, Lincoln, NE, USA).

### 2.5 RNA isolation, reverse transcription, and real‐time quantitative PCR

Total RNA was isolated from indicated cells using TRIzol reagent (Takara Bio, Dalian, China) and subsequently used for reverse transcription with a PrimeScript RT‐PCR reagent kit (Takara Bio). cDNA was subjected to real‐time quantitative polymerase chain reaction (RT‐qPCR) with an SYBR Green PCR kit (Takara Bio) on an ABI7500 Real‐time PCR system (Applied Biosystems, Inc., Austin, TX, USA). The primer sequences used in this study were shown as follows: SF3B1‐F, 5′‐GTGGGCCTCGATTCTACAGG‐3′; SF3B1‐R, 5′‐GATGTCACGTATCCAGCAAATCT‐3′; PPP2R5A‐F, 5′‐AGAGCCCTGATTTCCAGCCTA‐3′; PPP2R5A‐R, 5′‐TTTCCCATAAATTCGGTGCAGA‐3′; Myc‐F, 5′‐GTCAAGAGGCGAACACACAAC‐3′; Myc‐R, 5′‐TTGGACGGACAGGATGTATGC‐3′; ACTB‐F, 5′‐ACTCGTCATACTCCTGCT‐3′; ACTB‐R, 5′‐GAAACTACCTTCAACTCC‐3′. The 2‐^ΔΔCt^ method was used to calculate the relative expression of target genes. The ACTB gene was used as an internal control. For reverse transcriptase polymerase chain reaction (RT‐PCR), the primers used in this study were shown as follows: PPP2R5A‐F, GCCTAGCATTGCAAAACGAT; PPP2R5A‐R, GCAATGCAAAGCCATTGATA.

### 2.6 Immunohistochemical (IHC) analysis

Tissue sections from xenograft mouse models were fixed, embedded, and sectioned. IHC analysis was performed as described previously [34]. Primary antibodies against Ki‐67 (diluted at 1 : 400; Cell Signaling Technology, #9449) and SignalStain^®^ Boost IHC Detection Reagent (HRP, Mouse) (Cell Signaling Technology, #8125) were used.

### 2.7 Cell viability

Cell viability was detected by Cell Counting Kit‐8 (CCK‐8; Dojindo Molecular Technologies, Shanghai, China). In brief, PDAC cells were seeded into 96‐well plates at a density of 3,000 cells per well. At the indicated time, 10% (v/v) CCK‐8 reagent was added to each well prior to incubation at 37 °C for another 1 h. Then, absorbance at 450 nm was measured by an enzyme‐linked immunosorbent assay reader (PerkinElmer, Inc., Waltham, MA, USA).

### 2.8 Colony formation assay

For colony formation analysis, 500 viable cells were plated in 6‐well plates and maintained in a complete medium for 10–14 days. Once colonies were visible, they were fixed with 4% paraformaldehyde and stained with 0.5% crystal violet, and photographed. The experiment was repeated at least three times. The number of colonies was counted under an Olympus inverted light microscope (Olympus, Tokyo, Japan).

### 2.9 Glucose uptake and lactate production

As reported previously [35], the Glucose Colorimetric Assay Kit (Sigma‐Aldrich, MAK263) and Lactate Assay Kit (BioVision, Palo Alto, CA, USA, K607‐100) were used to determine glucose uptake and lactate production according to the manufacturer's protocols, respectively. Briefly, 3–5 × 10^5^ PDAC cells were seeded into 6‐well culture plates. Twenty‐four hours later, cell culture supernatants were collected after 24 h and subjected for analysis. The final level of glucose and lactate was normalized to total protein content as measured by the Pierce BCA Protein assay (Pierce Biotechnology, Rockford, IL, USA).

### 2.10 Measurement of extracellular acidification rate

The extracellular acidification rate (ECAR) was determined using the Seahorse Bioscience XF96 Extracellular Flux Analyzer (Seahorse Bioscience, Billerica, MA, USA). Experiments were performed according to the manufacturer's protocols. Seahorse XF Cell Glycolysis Stress Test Kit (Seahorse Bioscience) was used for ECAR measurement. In brief, 2 × 10^4^ cells per well were seeded in an XF96‐well plate. After baseline measurements, glucose, the oxidative phosphorylation inhibitor oligomycin, and the glycolytic inhibitor 2‐DG were sequentially injected into each well at indicated time points. Finally, data were assessed by Seahorse XF‐96 Wave software and ECAR is shown in mpH/minute.

### 2.11 Measurement of PP2A activity

PP2A activity was determined by PP2A Immunoprecipitation Phosphatase Assay Kit (Sigma‐Aldrich, 17‐313) according to the manufacturer’s protocol. In brief, Panc05.04 cells were treated with 1 μm FTY720 for 24 h and were then lysated by NP‐40 lysis buffer supplemented with protease inhibitor cocktail. Finally, immunoprecipitation of PP2A was conducted and its activity was assessed by dephosphorylation of the phosphopeptide (K‐R‐pT‐I‐R‐R).

### 2.12 Xenograft assay

Six‐week‐old male nude mice were obtained from the Chinese Academy of Sciences (Shanghai, China). Mice were randomly grouped with five mice in each group, and PDAC cells were subcutaneously in the lower back of nude mice. FTY‐720 was dissolved in water and administered intraperitoneally (3 mg·kg^−1^·day^−1^). All nude mice were sacrificed 1 month after injection. The tumor xenografts were separated and weighed. This study was approved by the Research Ethics Committee of Shanghai Jiao Tong University.

### 2.13 Statistical analysis

All data were presented as the mean ± SD from three independent experiments. Student's *t*‐test and one‐way analysis of variance (ANOVA) were used to compare differences between two groups and multiple groups, respectively. Tukey’s test was used for *post hoc* analysis following ANOVA. graphpad prism (GraphPad Software Inc., San Diego, CA, USA) was used for statistical analyses. The prognostic value of SF3B1 expression was conducted by the Kaplan–Meier method and analyzed by the log‐rank test. In all statistical analyses, *P* < 0.05 was considered statistically significant.

## 3 Results

### 3.1 SF3B1 mutation in human cancers

**Fig. 1:**
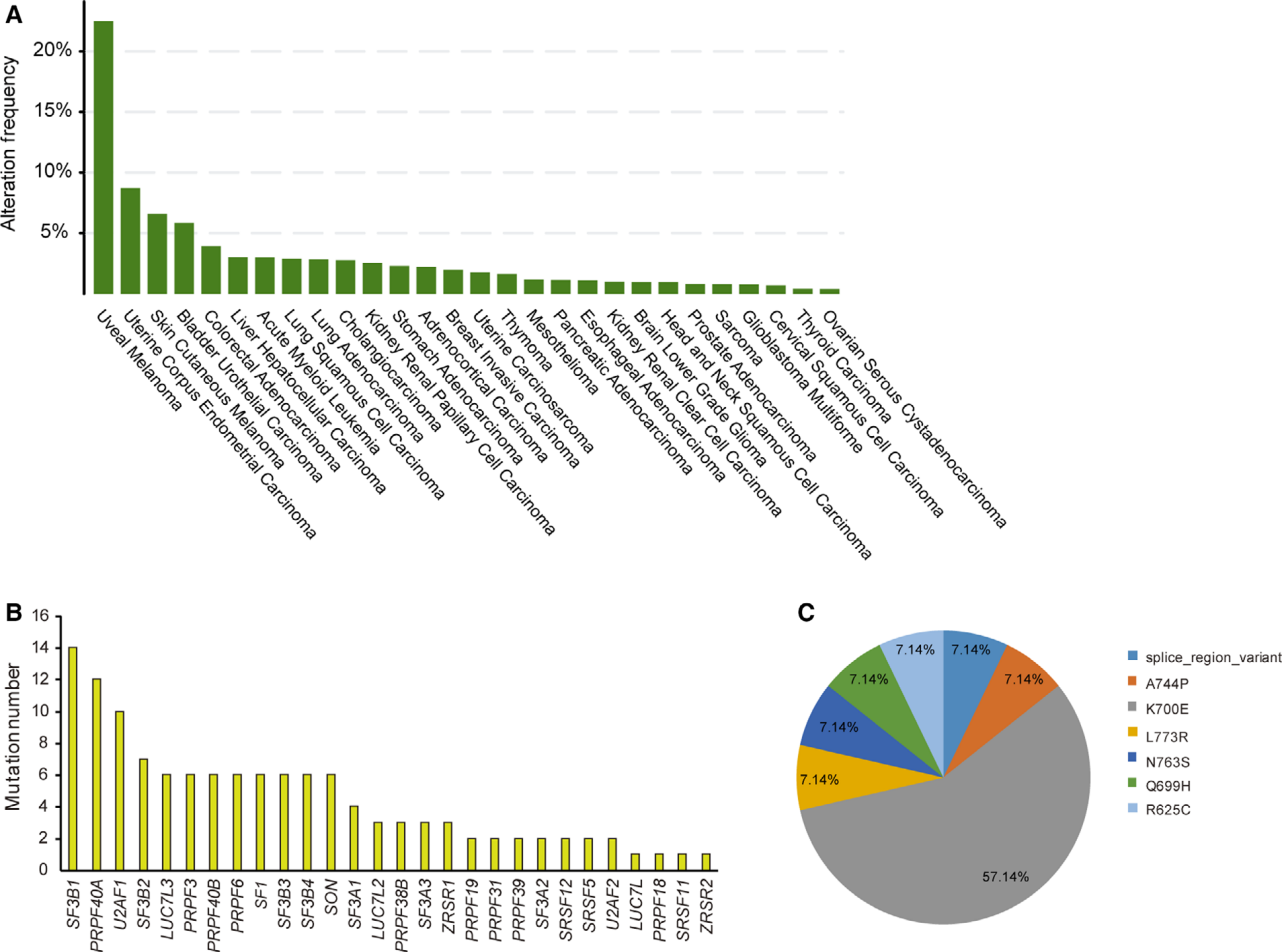
Mutation landscape of key splicing factors. (A) Pan‐cancer analysis of the mutation profile of SF3B1 in human cancers. Data were derived from the TCGA cohort. (B) The mutation frequency of key splicing factors. Data were derived from the ICGC database: PACA‐AU (431 cases) and PACA‐CA (252 cases). (C) Location and frequency of SF3B1 hot spot mutations. Data were derived from the ICGC database.

Previously, many studies observed that SF3B1 is commonly mutated in human cancers, especially MDS [36, 37]. By searching the cBioPortal database (http://www.cbioportal.org/), we found that SF3B1 had a high mutation frequency across multiple cancers, such as uveal melanoma and bladder cancer (Fig. 1A). In the TCGA cohort, however, only 3/179 PDAC samples harbored SF3B1 mutations (Fig. 1A). Furthermore, by data mining the PDAC cohort in the ICGC database, we revealed that SF3B1 had the highest mutation frequency among the 28 splicing factors analyzed (Fig. 1B). More interestingly, the SF3B1 K700E was the most frequent hot spot mutation with a frequency of 57.14% (Fig. 1C).

### 3.2 The effects of SF3B1 knockdown on PDAC cell proliferation

By real‐time qPCR and western blotting analysis, we observed that SF3B1 was universally expressed in PDAC cell lines. At mRNA level, MiaCapa‐2 and SW1990 cells had a lower SF3B1 expression in comparison with nonmalignant HPDE cells (Fig. S1B). Inconsistently, CFPAC‐1 and Panc05.04 had a higher protein level of SF3B1 compared with HPDE cells (Fig. S1B). Data from TCGA + GTEx showed that SF3B1 was not overexpressed in PDAC (*n* = 179) compared with normal pancreas tissues (*n* = 171) (Fig. S1C). Moreover, SF3B1 expression was comparable between SFB1 WT and SF3B1 MUT samples (Fig. S1D). Kaplan–Meier curves showed that SF3B1 expression was not associated with overall survival in PDAC patients (Fig. S1E).

**Fig. 2:**
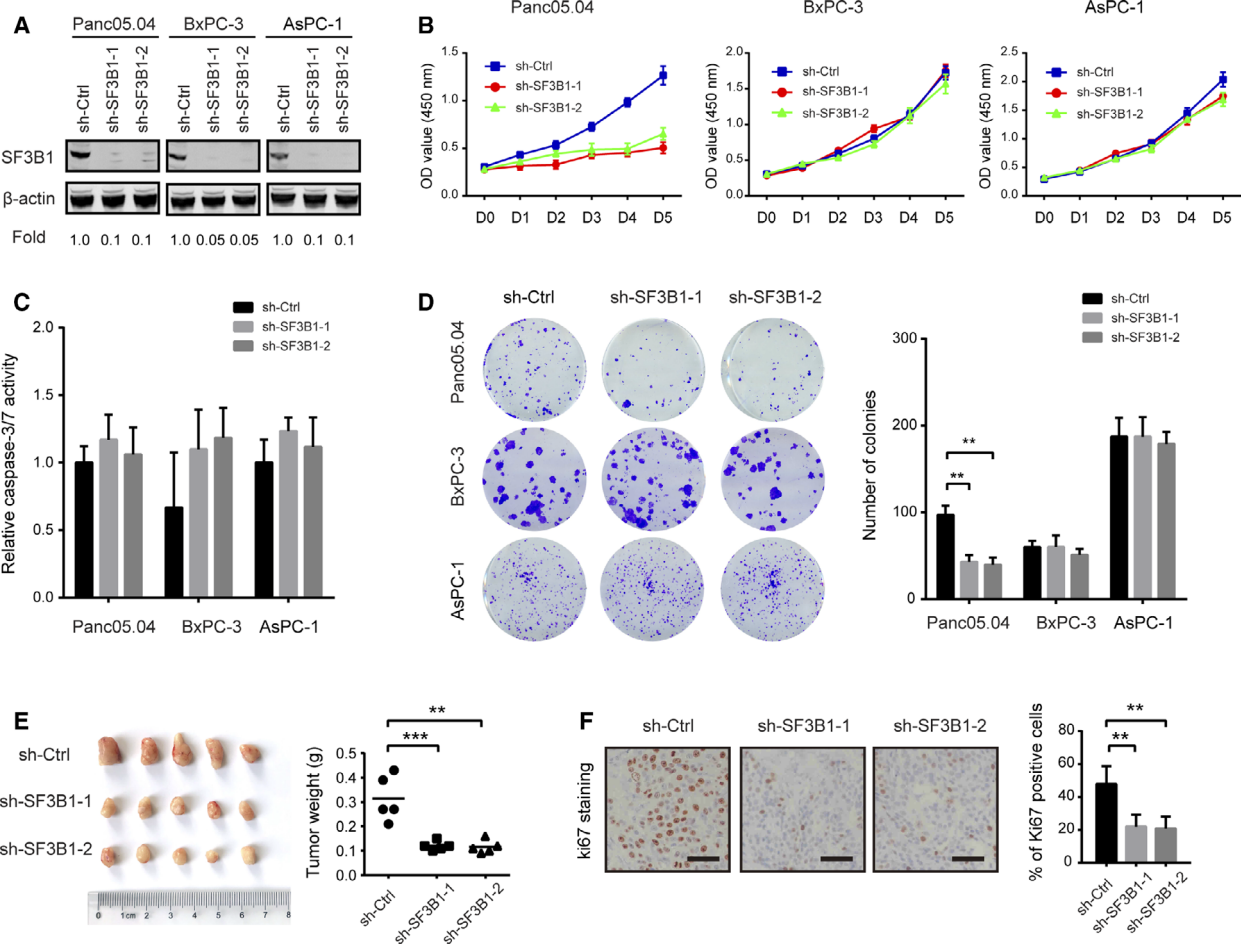
The effects of SF3B1 knockdown on PDAC cell proliferation. (A) The knockdown efficiency of SF3B1 in Panc05.04, BxPC3, and AsPC1 cells was analyzed by western blotting. (B) The effect of SF3B1 knockdown on Panc05.04, BxPC3, and AsPC1 cell proliferation was determined by CCK‐8 assay (*n* = 3). (C) The effect of SF3B1 knockdown on Panc05.04, BxPC3, and AsPC1 cell apoptosis was determined by caspase‐3/7 activity assay (*n* = 3). (D) The effect of SF3B1 knockdown on the colony formation ability of Panc05.04, BxPC3, and AsPC1 cells (*n* = 3). (E) Panc05.04‐sh‐Ctrl and Panc05.04‐sh‐SF3B1 cells were subcutaneously injected in nude mice; gross xenografts were shown, and tumor weight was analyzed. (F) IHC analysis showed representative Ki67 staining in Panc05.04‐sh‐Ctrl and Panc05.04‐sh‐SF3B1 xenograft tumor tissues. Scale bar: 50 μm. Data were presented as the mean ± SD; **P < 0.01; ***P < 0.001; statistical significance was calculated by Student’s *t*‐test.

To determine the cellular function of SF3B1 in PDAC, we genetically silenced SF3B1 in three cell lines, Panc05.04, BxPC3, and AsPC1. The SF3B1 K700E mutation was present in Panc05.04 cells, while BxPC3 and AsPC1 cell lines carried wild‐type SF3B1. As shown in Fig. 2A, SF3B1 protein expression was markedly downregulated by two shRNAs against SF3B1 in all three cell lines. By CCK‐8 assay, we noticed that SF3B1 knockdown significantly reduced the cell viability of Panc05.04 cells but not BxPC3 and AsPC1 cells (Fig. 2B). Of note, cell apoptosis was not affected by SF3B1 knockdown in all three cell lines (Fig. 2C). To observe the long‐term effect of SF3B1 knockdown, we performed plate colony formation assay. Similarly, a suppressive effect was only found in SF3B1‐mutant Panc05.04 cells (Fig. 2D). Moreover, we generated an *in vivo* xenograft model by subcutaneous injection of Panc05.04‐sh‐Ctrl and Panc05.04‐sh‐SF3B1 cells in nude mice. The results showed that xenografts from Panc05.04‐sh‐SF3B1 cells have reduced tumor weight (Fig. 2E) and positive staining for Ki67 (Fig. 2F) compared that from Panc05.04‐sh‐Ctrl cells.

### 3.3 SF3B1 K700E mutation plays a growth‐promoting role in PDAC

**Fig. 3:**
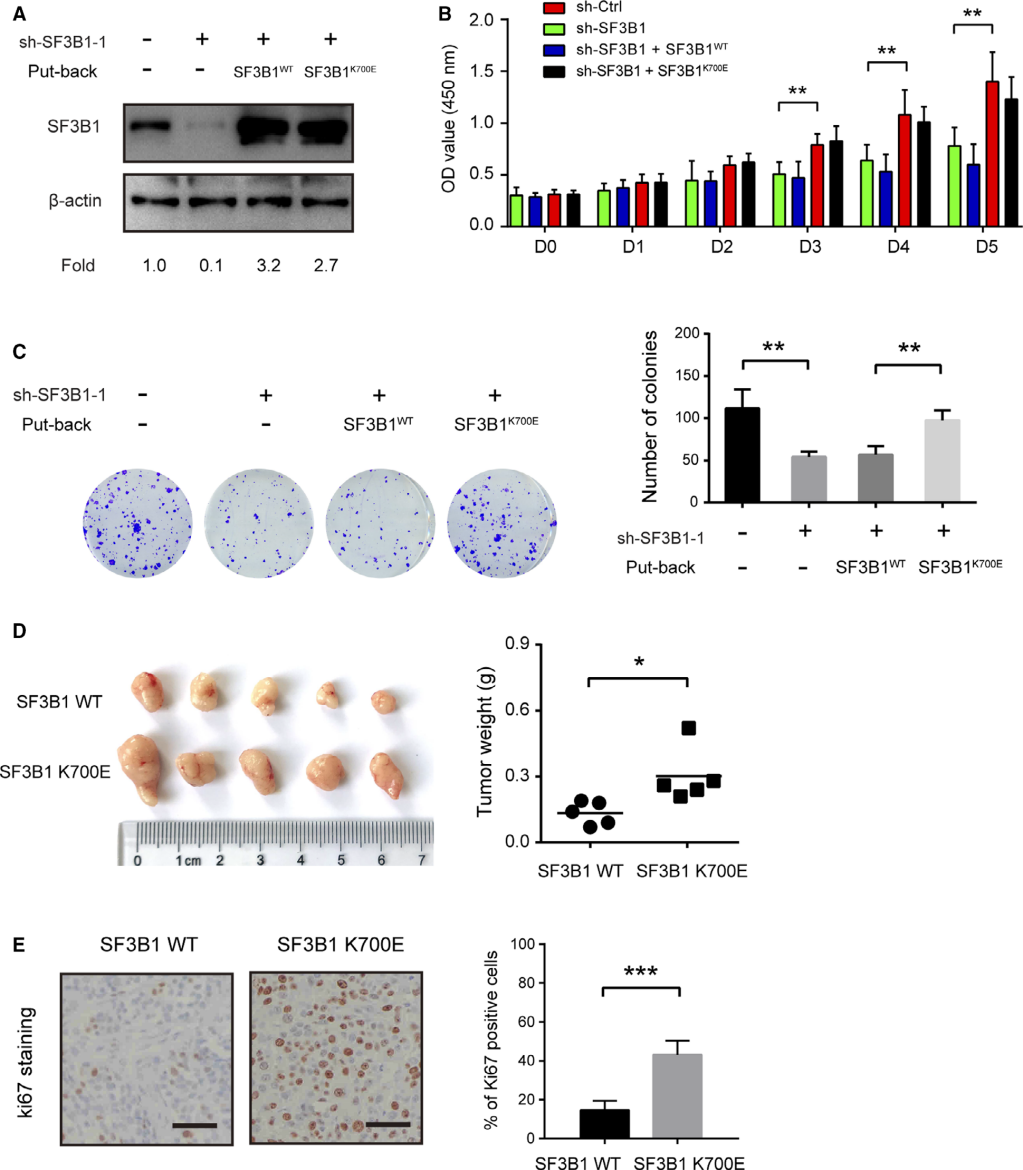
SF3B1 K700E mutation plays a growth‐promoting role in PDAC. (A) Panc05.04 cells stably knockdown SF3B1 and re‐express the shRNA‐resistant wild‐type or K700E mutant were established. SF3B1 knockdown efficiency and re‐expression were determined by western blotting. (B) CCK8 analysis for SF3B1‐WT or SF3B1‐K700E overexpression on the cell viability of Panc05.04‐sh‐SF3B1 cells (*n* = 5); (C) effect of SF3B1‐WT or SF3B1‐K700E overexpression on the colony formation ability of Panc05.04‐sh‐SF3B1 cells (*n* = 3). (D) SF3B1‐WT‐ or SF3B1‐K700E‐overexpressing Panc05.04‐sh‐SF3B1 cells were subcutaneously injected in nude mice; gross xenografts were shown, and tumor weight was analyzed. (E) IHC analysis showed representative Ki67 staining in SF3B1‐WT‐ and SF3B1‐K700E‐overexpressing Panc05.04‐sh‐SF3B1 xenograft tumor tissues. Scale bar: 50 μm. Data were presented as the mean ± SD; *P < 0.05, **P < 0.01, and ***P < 0.001 by one‐way ANOVA and Tukey’s multiple comparisons test (B, C) or Student’s *t*‐test (D, E).

To further investigate the effect of SF3B1 mutation on PDAC cells, we genetically silenced endogenous SF3B1 in three cell lines (BxPC3, AsPC1, and Panc05.04) by shRNA‐1 and re‐expressed shRNA‐resistant wild‐type SF3B1 (SF3B1^WT^) and the K700E mutant (SF3B1^K700E^). As shown in Fig. 3A and S3A ectopic expression of SF3B1^WT^ or SF3B1^K700E^ restored SF3B1 to a level comparable to endogenous SF3B1. Interestingly, re‐expression of SF3B1^K700E^ was sufficient to compromise the reduced cell viability and colony formation ability induced by SF3B1 knockdown in Panc05.04 cells, while SF3B1^WT^ was much less effective in restoring the cell proliferation of sh‐SF3B1 Panc05.04 cells (Fig. 3B,C). In sh‐SF3B1 BxPC3 and AsPC1 cells, ectopic expression of SF3B1 K700E but not SF3B1 WT also exhibited a promotive on the cell proliferation (Fig. S3A,B). To confirm the biological significance of SF3B1^K700E^ in tumor growth, we performed a xenograft experiment using SF3B1‐WT‐ or SF3B1‐K700E‐overexpressing sh‐SF3B1 cells. As a result, SF3B1‐K700E‐overexpressing Panc05.04 cells displayed tumor growth more seriously than the SF3B1‐WT‐overexpressing Panc05.04 cells (Fig. 3D). IHC analysis of Ki67 showed that tumor tissues from the SF3B1‐K700E‐overexpressing group had more positive staining than those from the SF3B1‐WT‐overexpressing group (Fig. 3E). Collectively, these findings suggest that the SF3B1 mutation is critically involved in cell proliferation and tumor growth of PDAC.

### 3.4 SF3B1 mutation promotes glycolytic metabolism in PDAC cells

**Fig. 4:**
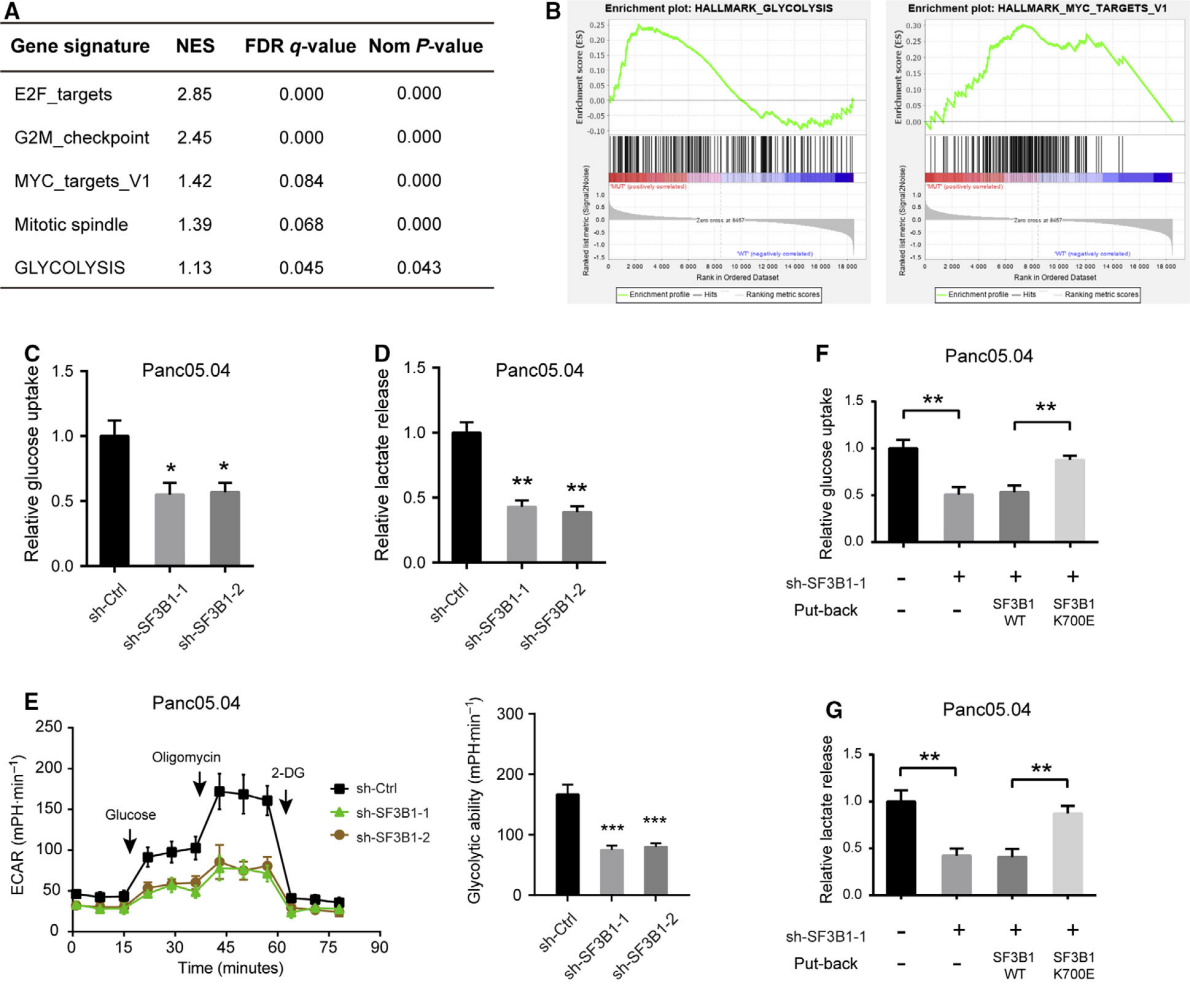
SF3B1 mutation promotes glycolytic metabolism in PDAC cells. (A) Gene set enrichment analysis based on Hallmark gene sets revealed gene sets related to SF3B1 mutation; false discovery rate was set at 0.25; NES indicates normalized enrichment score. (B) GSEA plot of MYC target and glycolysis related to SF3B1 mutation. (C‐E) Measurement of glucose uptake, lactate release, and ECAR in sh‐Ctrl and sh‐SF3B1 Panc05.04 cells (*n* = 3). (F) Effects of SF3B1‐WT or SF3B1‐K700E overexpression on the glucose uptake of Panc05.04‐sh‐SF3B1 cells (*n* = 3). (G) Effects of SF3B1‐WT or SF3B1‐K700E overexpression on the lactate release of Panc05.04‐sh‐SF3B1 cells (*n* = 3). Data were presented as the mean ± SD; *P < 0.05, **P < 0.01, and ***P < 0.001 by one‐way ANOVA and Tukey’s multiple comparisons test (C‐E) or Student’s *t*‐test (F, G).

To determine the mechanism by which SF3B1 mutation promotes tumor growth in PDAC, we performed gene set enrichment analysis by utilizing RNA sequencing data from TCGA. Among 179 PDAC samples, three samples had SF3B1 mutations. GSEA revealed that the SF3B1 mutation was closely associated with five gene signatures, including E2F_targets, G2M_checkpoint, MYC_targets_V1, mitotic spindle, and glycolysis (Fig. 4A,B). These pathways indicate that SF3B1 mutation may contribute to cell cycle progression and tumor glucose metabolism. To verify the potential effect of SF3B1 mutation on the glycolytic phenotype of PDAC cells, we analyzed glucose and lactate levels in sh‐Ctrl and sh‐SF3B1 PDAC cells. Indeed, knockdown of SF3B1 significantly inhibited cell glucose uptake and lactate release in Panc05.04 cells (Fig. 4C,D), but not in BxPC3 and AsPC1 cells (Fig. S2A,B). Using Seahorse XF Analyzers, we confirmed that ECAR was reduced by SF3B1 knockdown in SF3B1‐mutant PDAC cells (Figs 4E and Fig. S2C). Moreover, re‐expression of SF3B1^K700E^ but not SF3B1^WT^ was sufficient to restore the SF3B1 knockdown‐mediated reduction in glucose uptake and lactate production in Panc05.04 cells (Fig. 4F,G). Although glycolytic ability of BxPC3 and AsPC1 cells was not altered by SF3B1 knockdown, ectopic expression of SF3B1K700E but not SF3B1WT significantly promoted glucose uptake and lactate production (Fig. S4C). Taken together, these data indicate that the SF3B1 mutation may play a role in the regulation of glycolytic metabolism in PDAC.

### 3.5 SF3B1 mutations increase c‐Myc expression and regulate PPP2R5A splicing in PDAC cells

**Fig. 5:**
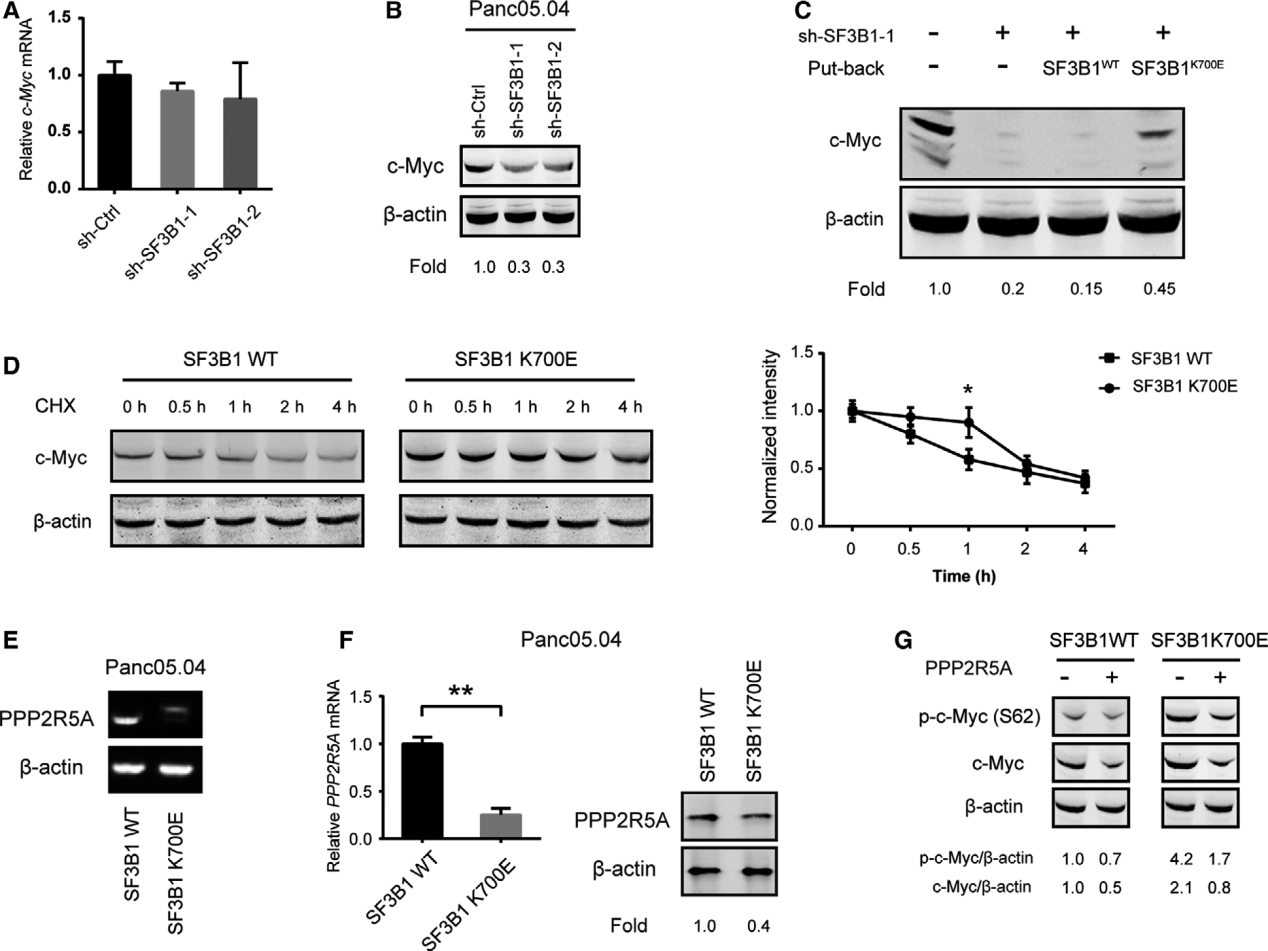
SF3B1 mutations increase c‐Myc expression and regulate PPP2R5A splicing in PDAC cells. (A) Real‐time qPCR analysis of the effect of SF3B1 knockdown on c‐Myc mRNA expression in Panc05.04 cells (*n* = 3); statistical significance was calculated by Student’s *t*‐test. (B) Western blotting analysis of the effect of SF3B1 knockdown on c‐Myc protein expression in Panc05.04 cells; error bars indicate technical replicates. (C) Western blotting analysis of the effect of SF3B1‐WT or SF3B1‐K700E overexpression on the c‐Myc protein expression in Panc05.04‐sh‐SF3B1 cells. (D) SF3B1‐WT‐ or SF3B1‐K700E‐overexpressing Panc05.04‐sh‐SF3B1 cells were treated with 100 μg·mL^−1^ CHX for the indicated times; then, the cell extracts were harvested and subjected to western blotting analysis of c‐Myc protein expression. (E) Representative RT‐PCR result of aberrantly spliced transcripts of PPP2R5A in SF3B1‐WT‐ or SF3B1‐K700E‐overexpressing Panc05.04‐sh‐SF3B1 cells. (F) Real‐time qPCR and western blotting analysis of the effect of SF3B1‐WT or SF3B1‐K700E on PPP2R5A expression in Panc05.04‐sh‐SF3B1 cells. (G) Western blotting analysis of the effect of PPP2R5A restoration on the total and phosphorylation levels of c‐Myc in SF3B1‐WT‐ or SF3B1‐K700E‐overexpressing Panc05.04‐sh‐SF3B1 cells. Data were presented as the mean ± SD; *P < 0.05; **P < 0.01. Student’s *t*‐test was used to compare differences between two groups.

It is well known that c‐Myc is a key transcription factor for aerobic glycolysis [38]. Interestingly, we found that the SF3B1 mutation was associated with alteration of c‐Myc signaling in PDAC (Fig. 4A,B). By real‐time qPCR, we failed to observe changes in c‐Myc mRNA levels upon SF3B1 knockdown in Panc05.04 cells (Fig. 5A). However, SF3B1 knockdown resulted in a marked downregulation of c‐Myc protein expression in Panc05.04 cells (Fig. 5B). Moreover, re‐expression of SF3B1K700E in sh‐SF3B1 Panc05.04 cells largely restored c‐Myc protein expression (Fig. 5C), indicating that the regulation of c‐Myc expression by SF3B1 might occur via post‐transcriptional and/or post‐translational mechanisms. To uncover whether SF3B1 mutation stabilizes c‐Myc protein, we detected c‐Myc protein level in SF3B1‐WT‐ or SF3B1‐K700E‐overexpressing Panc05.04‐sh‐SF3B1 cells in the presence of 100 μg·mL^−1^ cycloheximide (CHX). As shown in Fig. 5D, shSF3B1 cells with restored SF3B1K700E had significant increased level of c‐Myc at every time points compared with shSF3B1 control cells after CHX was treated to arrest protein synthesis. Notably, SF3B1‐K700E‐expressing cells showed significant stability of c‐Myc protein compared with SF3B1‐WT‐expressing at 1 h after CHX treatment (Fig. 5D). Previously, it was well documented that aberrant splicing of PPP2R5A induced by SF3B1 mutation leads to MYC activation via post‐translational regulation. To test this possibility in PDAC, we analyzed PPP2R5A expression in the SF3B1‐mutant Panc05.04 cells. RT‐PCR analysis showed that aberrant splicing of PPP2R5A was present in SF3B1‐K700E‐overexpressing sh‐SF3B1‐Panc05.04 cells (Fig. 5E). Western blotting and real‐time qPCR revealed that both PPP2R5A mRNA and protein expression were significantly suppressed by the SF3B1 K700E mutation (Fig. 5F). PPP2R5A acts as a regulatory subunit of the major serine/threonine PP2A protein complex, which can dephosphorylate c‐Myc at serine 62 and lead to c‐Myc degradation. By western blotting analysis, we noticed a significant increase in phosphorylated c‐Myc levels in SF3B1^K700E^ cells compared with SF3B1^WT^ cells. Ectopic expression of PPP2R5A largely abrogated the increased phosphorylation level of c‐Myc induced by SF3B1‐K700E in sh‐SF3B1‐Panc05.04 cells (Fig. 5G). Taken together, aberrant PPP2R5A splicing induced by SF3B1 mutation might contribute to c‐Myc activation and subsequent glycolytic metabolism and tumor growth.

### 3.6 Pharmacological activation of PP2A suppresses cell proliferation and aerobic glycolysis in SF3B1‐mutant PDAC cells

**Fig. 6:**
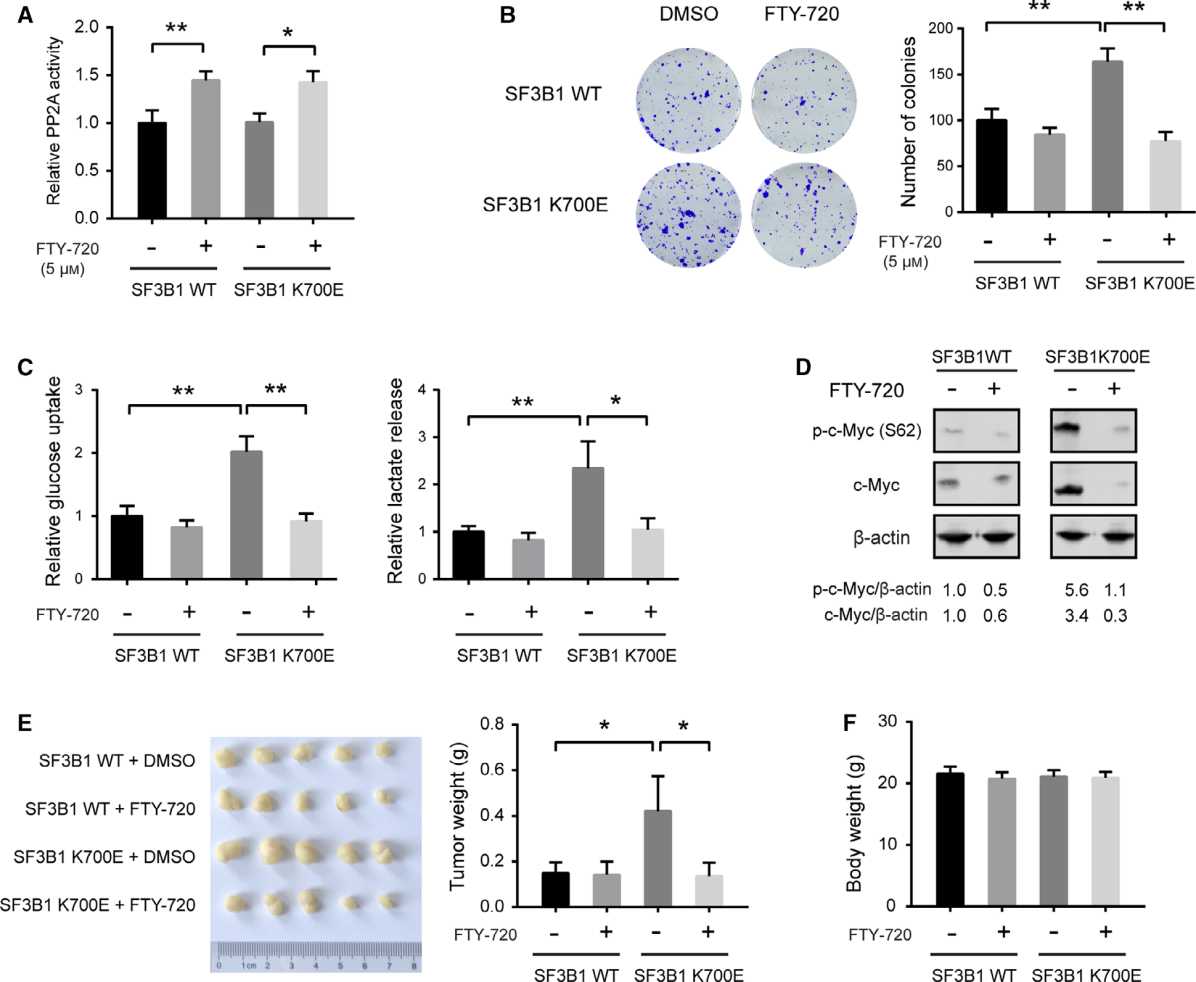
Pharmacological activation of PP2A suppresses cell proliferation and aerobic glycolysis in SF3B1‐mutant PDAC cells. (A) Measurement of PP2A activity after FTY‐720 treatment for 24 h in SF3B1‐WT‐ or SF3B1‐K700E‐overexpressing Panc05.04‐sh‐SF3B1 cells (*n* = 3). (B) The effect of PP2A activation by FTY‐720 on the colony formation ability of SF3B1‐WT‐ or SF3B1‐K700E‐overexpressing Panc05.04‐sh‐SF3B1 cells (*n* = 3). (C) The effect of FTY‐720 treatment on the glucose uptake and lactate release of SF3B1‐WT‐ or SF3B1‐K700E‐overexpressing Panc05.04‐sh‐SF3B1 cells. (D) Western blotting analysis of the effect of FTY‐720 treatment on the total and phosphorylation levels of c‐Myc in SF3B1‐WT‐ or SF3B1‐K700E‐overexpressing Panc05.04‐sh‐SF3B1 cells. (E) SF3B1‐WT‐ or SF3B1‐K700E‐overexpressing Panc05.04‐sh‐SF3B1 cells were subcutaneously injected in nude mice; tumor growth was monitored in the presence of FTY‐710 treatment (3 mg·kg^−1^·day^−1^); gross xenografts were shown, and tumor weight was analyzed. (F) Body weight of mice used in (E). Data were presented as the mean ± SD; *P < 0.05; **P < 0.01; statistical significance was calculated by one‐way ANOVA and Tukey’s multiple comparisons test.

The immune modulator FTY720 is also an FDA‐approved oral PP2A activator [39]. From a therapeutic point of review, we tested the potential tumor‐suppressive role of FTY720 in Panc05.04 cells. As shown in Fig. 6A,B, PP2A activation by FTY‐720 inhibited the colony formation ability of SF3B1^WT^ Panc05.04 cells, and this inhibitory role was further boosted in SF3B1^K700E^ Panc05.04 cells. Moreover, FTY‐720 treatment led to glycolytic inactivation in SF3B1^K700E^ Panc05.04 cells as demonstrated by the downregulation of glucose uptake and lactate release (Fig. 6C). Consistently, FTY‐720 treatment significantly decreased the level of total and phosphorylated c‐Myc in SF3B1^K700E^ Panc05.04 cells (Fig. 6D). Furthermore, the *in vivo* effects of FTY‐720 were tested in a xenograft assay. The result showed that FTY‐720 inhibited tumor growth of SF3B1‐K700E‐overexpressing but not SF3B1‐WT Panc05.04‐sh‐SF3B1 cells (Fig. 6E). Notably, the body weight of mice treated with FTY‐720 remained unaltered, suggesting that FTY‐720 is nontoxic *in vivo* (Fig. 6F). Collectively, these results suggest that SF3B1 K700E mutation leads to the aberrant splicing of PPP2R5A and the increased expression of c‐Myc, which leads to an enhanced Warburg effect and subsequent tumor growth in PDAC.

## 4 Discussion

Accumulating evidence suggests that dysregulation or mutations in SF3B1 represent driver events in many malignancies. SF3B1 has been reported to be overexpressed in prostate cancer [40], hepatocellular carcinoma [41], and breast cancer. In breast cancer, overexpressed SF3B1 is associated with lymph node metastasis and SF3B1 knockdown inhibits breast cancer cell proliferation, invasion, and migration via aberrant splicing [42]. Moreover, pharmacological inhibition of SF3B1 with Jerantinine A induces significant tumor‐specific cell death and a significant increase in unspliced pre‐mRNAs [43]. Apart from expression dysregulation, abundant data have focused on studies of mutations in SF3B1 [28, 44]. In this study, we report that hot spot mutations in SF3B1 lead to a metabolic reprogramming and growth‐promoting effects and induce vulnerability to PP2A activators in PDAC cells.

PDAC is a highly lethal malignancy characterized by a high frequency of mutations in classical oncogenes and tumor suppressors, such as *KRAS*, *TP53*, *CDKN2A*, and *SMAD4*. As reported by Christopher J. Scarlett *et al*., SF3B1 is one of the most significantly mutated genes in PDAC [26]. However, the clear biological functions of SF3B1 mutation in PDAC tumorigenesis have not been demonstrated. In this study, we revealed a tumor‐promoting role of SF3B1 mutation in PDAC cells via loss‐of‐function and gain‐of‐function studies. Genetic silencing of SF3B1 only displayed a tumor‐suppressive effect in a PDAC cell line harboring SF3B1 mutation but not wild‐type SF3B1, suggesting a change‐of‐function of SF3B1 mutation in PDAC. PDAC is characterized by severe desmoplasia and a tumor microenvironment with hypoxia and a lack of nutrients. However, most of our observations are based on *in vitro* cell experiments, which were conducted under nonphysiological nutrient conditions (high saturating glucose) and oxygen tension. Moreover, our *in vivo* findings were noticed in nude athymic mice and did not reflect the complicated immune response in the tumor microenvironment. Thus, the cellular functions of SF3B1 mutations in PDAC warrant further investigation.

SF3B1 mutations play different roles in the biological processes of different tumor types. The high mutation rate of SF3B1 in hematological tumors strongly suggests its tumor‐driving effect. SF3B1 mutations reduce the response of CLL patients to DNA damage, leading to rapid disease progression and poor prognosis in SF3B1 mutant CLL patients [45]. In contrast, Furney and Harbour et al. sequenced SF3B1 mutations in 117/105 uveal melanoma patients, respectively, and found that the SF3B1 mutations were all associated with a lower stage and a better prognosis [27, 46]. The different splicing events induced by SF3B1 mutations might contribute to diverse biological processes and clinical outcomes. Previously, several studies have documented the molecular mechanism underlying SF3B1‐mediated oncogenic activities. It is well documented that SF3B1 mutations facilitate usage of aberrant branch point residues, which commonly manifests in transcripts bearing aberrant intron‐proximal 3′splice site (3′ss). Two research teams performed transcriptome sequencing of SF3B1 mutation and wild‐type MDS and found that the downregulation of SLC25A37 alternative splicing leads to iron overload in cells. At the same time, alternative splicing of MDS pathogenic genes including ASXL1, CBL, ALAS2, ABCB7, PRRF8, and HNRNPD was discovered [47, 48]. In breast cancer, SF3B1 mutations induce missplicing‐associated downregulation of the serine synthesis pathway enzyme PHGDH and decrease mitochondrial respiration [49]. In prostate cancer, Liu et al. recently showed that mutations in SF3B1 promote decay of transcripts encoding the protein phosphatase 2A subunit PPP2R5A [25].

In the current study, we confirmed the molecular mechanisms of SF3B1 mutation in the regulation of the PPP2R5A‐PP2A‐c‐Myc axis in PDAC. Importantly, this finding for the first time couples SF3B1 mutation to reprogrammed glucose metabolism in PDAC. Interestingly, specific inhibitors of SF3B1, such as Spliceostain A and Pladienolide B, can change the splicing of MCL‐1, CDK, and VEGF, and inhibit the proliferation of many tumors including MDS and small‐cell lung cancer [50, 51]. Because these inhibitors have very low toxicity to normal cells, this indicates that SF3B1 can be used as a potential therapeutic target for PDAC treatment.

## 5 Conclusions

Our findings revealed the contribution of SF3B1 in PDAC and have identified a novel function of SF3B1 mutation in regulating the Warburg effect. Importantly, SF3B1 mutation was closely associated with the activity of the PP2A complex, indicating that targeting the SF3B1–PP2A–c‐Myc axis in combination with the first‐line chemotherapy regimen may improve the treatment effect for SF3B1‐mutant PDAC patients.

## Conflict of interest

The authors declare no conflicts of interest.

## Author contributions

WL, S‐HJ, and Y‐WS conceived the study plan. J‐YY, Y‐MH, M‐WY, and YS performed the experiments, analyzed the data, and finished the manuscript writing. D‐JL, X‐LF, L‐YT, R‐ZH, J‐FZ, and RH performed the *in vivo* experiments. J‐YY and Y‐MH contributed to data interpretation. All authors discussed the results and reviewed the manuscript for submission.

### Peer Review

The peer review history for this article is available at https://publons.com/publon/10.1002/1878‐0261.12970.

## Supporting information


**Fig. S1**. Expression pattern and prognostic value of SF3B1 in PDAC.
**Fig. S2**. Effects of SF3B1 knockdown on the glycolytic metabolism of AsPC1 and BxPC3 cells.
**Fig. S3**. SF3B1 mutation promotes cell proliferation and glycolytic metabolism in PDAC cells.
**Fig. S4**. SF3B1 mutations increase c‐Myc expression in PDAC cells.
**Table S1**. Cancer cell line information.

## Data Availability

The data that support the findings of this study are available from the corresponding author (shjiang@shsci.org) upon reasonable request.
