# Supplementary material for: SF3B1 mutation in pancreatic cancer contributes to aerobic glycolysis and tumor growth through a PP2A–c‐Myc axis
Source: Mol Oncol. 2021 May 3;15(11):3076–90. doi: 10.1002/1878-0261.12970 (PMC8564647; doi:10.1002/1878-0261.12970)
Supplement: Supplementary file 1 — Fig. S1. Expression pattern and prognostic value of SF3B1 in PDAC. Fig. S2. Effects of SF3B1 knockdown on the glycolytic metabolism of AsPC1 and BxPC3 cells. Fig. S3. SF3B1 mutation promotes cell proliferation and glycolytic metabolism in PDAC cells. Fig. S4. SF3B1 mutations increase c‐Myc expression in PDAC cells. Table S1. Cancer cell line information. [file MOL2-15-3076-s001.docx]

**Supplementary information**


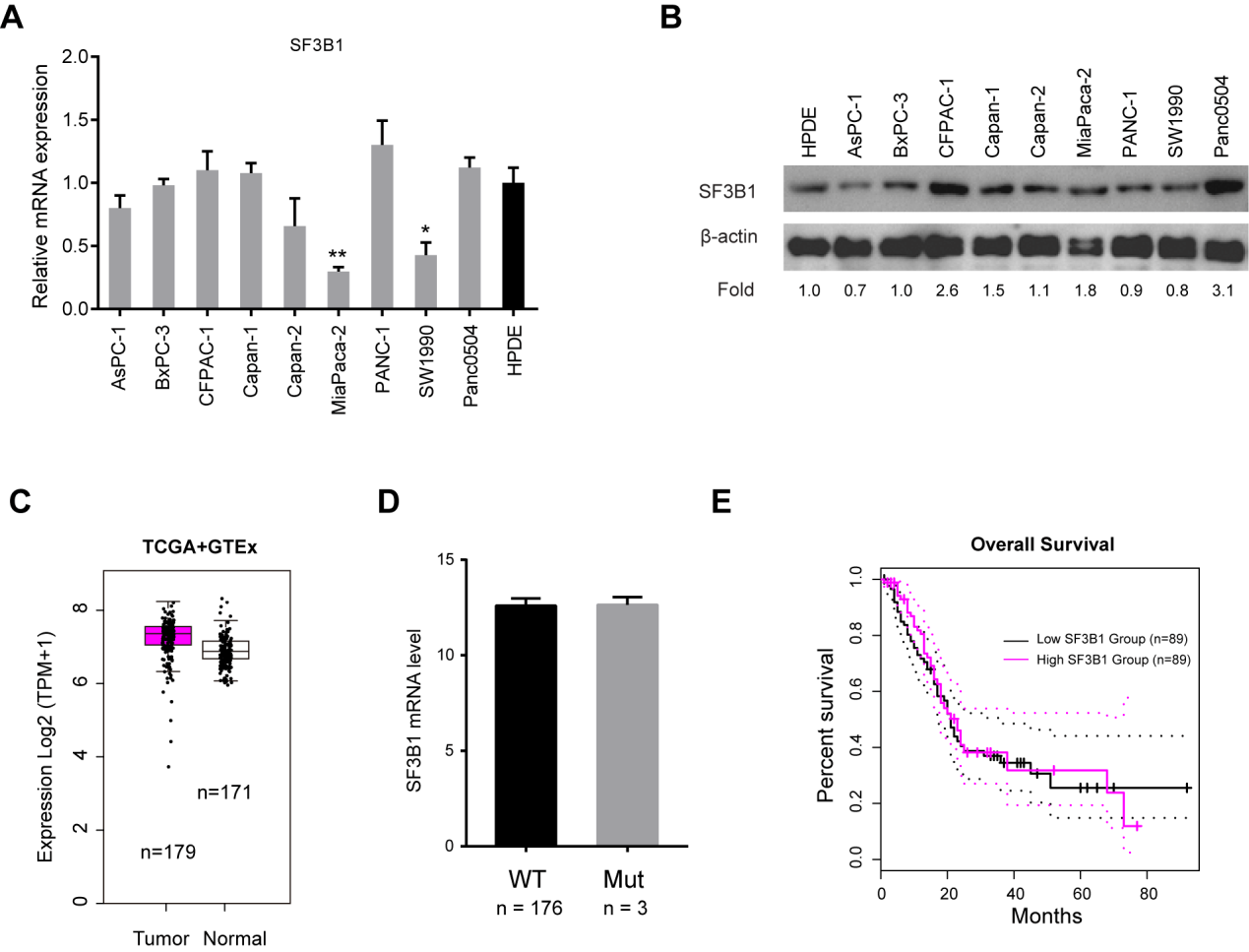


**Supplementary Figure 1. Expression pattern and prognostic value of SF3B1 in PDAC.** (A) Real-time qPCR analysis of SF3B1 mRNA levels in PDAC cell lines (n = 3). (B) Western blotting analysis of SF3B1 protein levels in PDAC cell lines (n = 3). (C) Comparison of SF3B1 expression level in PDAC tumor tissue samples and normal pancreas cases. Data were derived from TCGA cohort and GTEx. (D) Comparison of SF3B1 expression level in SF3B1 WT and SF3B1 MUT PDAC tumor tissue samples. (E) Kaplan-Meier graphs showing the association of SF3B1 expression with PDAC patients' survival. The median expression value of SF3B1 was used as a cutoff; the prognostic value was analyzed by the log-rank test; the dotted line represents the 95% confidence interval. HR: hazard ratio.


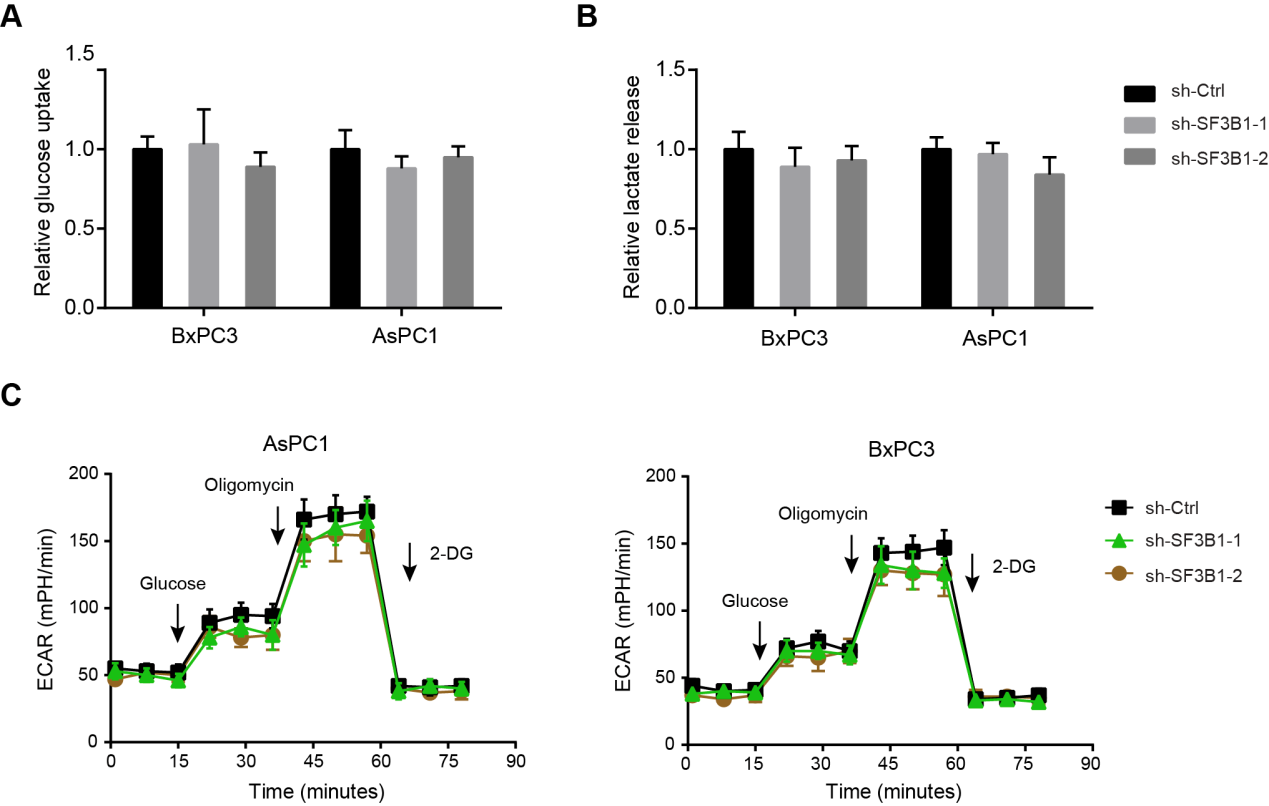


**Supplementary Figure 2. Effects of SF3B1 knockdown on the glycolytic metabolism of AsPC1 and BxPC3 cells.** (A) Measurement of glucose uptake in sh-Ctrl, sh-SF3B1-1 and sh-SF3B1-2 AsPC-1 and BxPC-3 cells (n = 3). (B) Measurement of lactate level in sh-Ctrl, sh-SF3B1-1 and sh-SF3B1-2 AsPC-1 and BxPC-3 cells (n = 3). (C) Detection of the extracellular acidification rate (ECAR) in sh-Ctrl, sh-SF3B1-1 and sh-SF3B1-2 AsPC-1 and BxPC-3 cells (n = 3). Statistical significance was calculated by Student’s t-test.

**
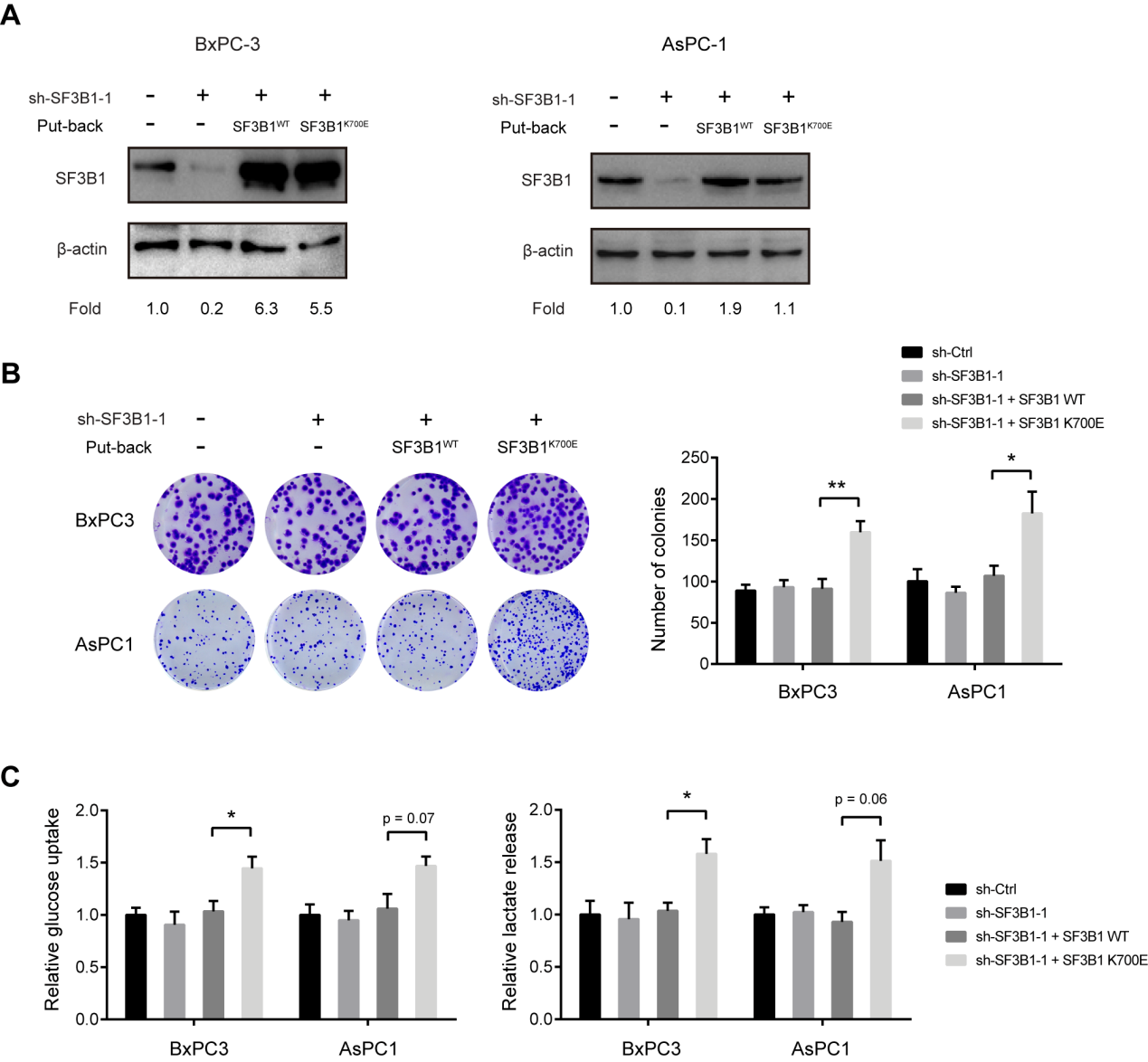
**

**Supplementary Figure 3. SF3B1 mutation promotes cell proliferation and glycolytic metabolism in PDAC cells.** (A) BxPC-3 and AsPC-1 stably knockdown SF3B1 and re-express the shRNA-resistant wild-type or K700E mutant were established. SF3B1 knockdown efficiency and re-expression were determined by western blotting. (B) Effect of SF3B1-WT or SF3B1-K700E overexpression on the colony-formation ability of sh-SF3B1 BxPC-3 and AsPC-1 cells (n = 3). (C) Effect of SF3B1-WT or SF3B1-K700E overexpression on the glucose uptake and lactate production of sh-SF3B1 BxPC-3 and AsPC-1 cells (n = 3). **P* < 0.05; ***P* < 0.01; statistical significance was calculated by Student’s t-test.

**
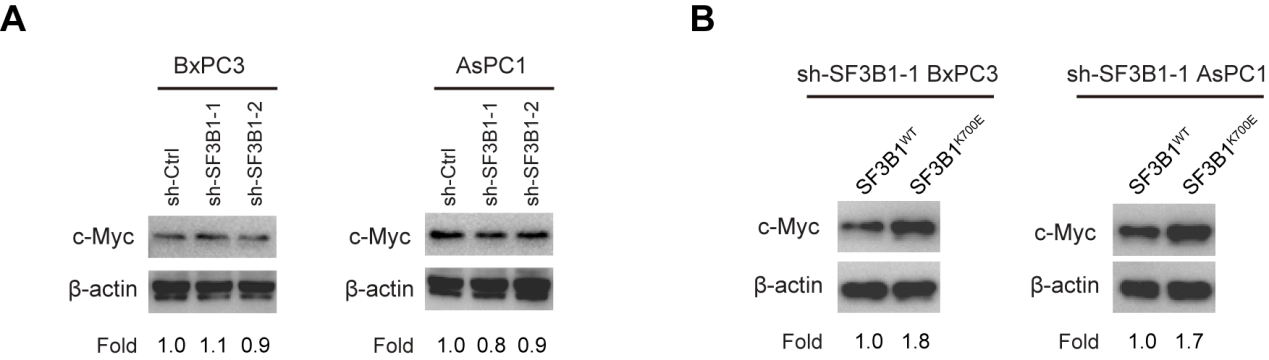
**

**Supplementary Figure 4. SF3B1 mutations increase c-Myc expression in PDAC cells.** (A) Western blotting analysis of the effect of SF3B1 knockdown on c-Myc protein expression in BxPC3 and AsPC1 cells. (B) Western blotting analysis of the effect of SF3B1-WT or SF3B1-K700E overexpression on the c-Myc protein expression in sh-SF3B1-1 BxPC3 and AsPC1 cells.

**Supplementary table 1: Cancer cell line information**

| **Cell line** | **Age** | **Sex** | **Derivation** | **Metastasis** | **Differentiation** | ***KRAS* mutation** |
| --- | --- | --- | --- | --- | --- | --- |
| AsPC-1 | 62 | Female | Ascites | Yes | Poor | 12 Asp |
| BxPC-3 | 61 | Female | Primary tumor | No | Moderate to poor | WT |
| Capan-1 | 40 | Male | Liver metastasis | Yes | Well | 12 Val |
| Capan-2 | 56 | Male | Primary tumor | No | Well | 12 Val |
| CFPAC-1 | 26 | Male | Liver metastasis | Yes | Well | 12 Val |
| MIAPaCa-2 | 65 | Male | Primary tumor | ND | Poor | 12 Cys |
| PANC-1 | 56 | Female | Primary tumor | Yes | Poor | 12 Asp |
| Panc05.04 | 77 | Female | Primary tumor | No | Well | 12 Asp |
| SW1990 | 56 | Male | Spleen metastasis | Yes | Moderate to poor | 12 Val |
